# The Progress of Anti-HBV Constituents from Medicinal Plants in China

**DOI:** 10.1007/s13659-018-0178-6

**Published:** 2018-07-05

**Authors:** Chang-An Geng, Ji-Jun Chen

**Affiliations:** 1 State Key Laboratory of Phytochemistry and Plant Resources in West China, Kunming Institute of Botany, Chinese Academy of Sciences, No. 132 Lanhei Road, Kunming, 650201 China; 2 Yunnan Key Laboratory of Natural Medicinal Chemistry, Kunming, 650201 China; 3 University of Chinese Academy of Sciences, Beijing, 100049 China

**Keywords:** Hepatitis B virus, Anti-HBV activity, Medicinal plants, HBsAg, HBeAg, HBV DNA

## Abstract

**Abstract:**

Hepatitis B virus (HBV) infection causing acute and chronic hepatitis is a serious problem worldwide, whereas the current treatment methods are unsatisfactory. Traditional Chinese herbs that have long been used for medicinal purposes are fascinating sources for novel anti-HBV candidates. This paper summarizes the progress of anti-HBV constituents from diverse medicinal plants in China to provide information for searching new anti-HBV drugs from natural sources.

**Graphical Abstract:**

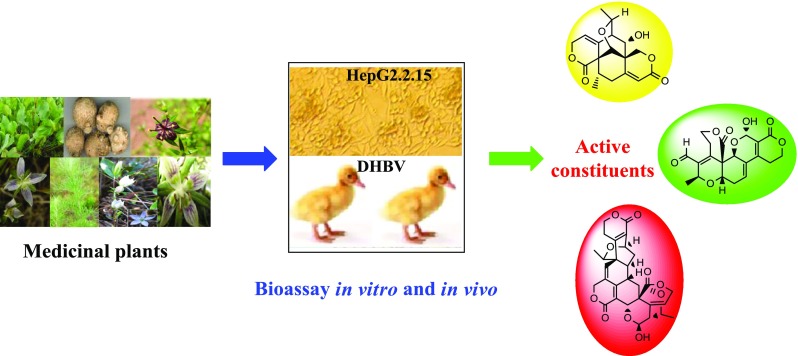

## Introduction

Hepatitis B infection caused by the hepatitis B virus (HBV) can become either acute or chronic diseases. HBV infections are among the top ten causes of death, and lead to 0.89 million deaths every year. It is estimated that 257 million people are living with chronic HBV infection. China is one of the high prevalence areas accounting for one-third of the infections. The major complications of chronic hepatitis B (CHB) are cirrhosis and hepatocellular carcinoma (HCC) [1, 2]. Hepatitis B vaccination is effective in preventing HBV infections; however, it is invalid to those who have already been infected with HBV. Interferons and nucleoside analogs are the most common drugs for CHB in clinic. Interferons (INF-α and PegIFN-α) take effects by enhancing the immune system of patients, but their application is limited by the low curing rate and serious side effects. Nucleoside analogues (mainly lamivudine, adefovir dipivoxil, telbivudine, entecavir, tenofovir disoprox and clevudine) can inhibit HBV replication by targeting on viral DNA polymerase; however, the drug-resistance and relapse are challenging after long-term treatment [3–5]. Currently, there is no cure for CHB, and new treatment methods with unprecedented targets are urgently needed. Traditional Chinese herbs that have long been used for medicinal purpose in China are fascinating sources for anti-HBV candidates [6, 7]. This paper summarized the progress of anti-HBV constituents from medicinal plants in China.

## Active Constituents from Medicinal Plants

According to the traditional Chinese medicine (TCM) theory, CHB is characteristic with the syndrome of liver stagnation and spleen deficiency. Thus, traditional Chinese herbs with the properties of “clearing heat removing dampness” and “promoting blood circulation and removing blood stasis” are preferable in searching anti-HBV candidates.

### *Artemisia* Plants

Plants of *Artemisia* contain about 300 species mainly present in the temperate, cold temperate and subtropical regions of Asia, Europe and North America. One hundred and eighty six Species and 44 variants are distributed in China, many of which are used as medicinal herbs. Yin-Chen is a famous TCM that has been used to treat jaundice in China for thousands of years. The first application of Yin-Chen could be traced back to the first Chinese dispensatory, “Shen-Nong-Ben-Cao-Jing”. Two species, namely *Artemisia capillaris* (Yinchenhao) and *Artemisia scoparia* (Binhao), are documented in Chinese Pharmacopoeia as the authentic resources of Yin-Chen. According to the bioassay on HepG 2.2.15 cell line in vitro, the 90% ethanol extract of *A. capillaris* exhibited activity against the secretions of HBsAg and HBeAg, and HBV DNA replication with IC_50_ value of 460.33 μg/mL (SI = 1.4), 295.31 μg/mL (SI = 2.5) and 49.13 μg/mL (SI = 13.4), respectively. Detailed LCMS analyses on the active part showed the presence of a series of chlorogenic acids. Subsequent isolation gave rise to chlorogenic acid (**1**), cryptochlorogenic acid (**2**), neochlorogenic acid (**3**), 3,5-dicaffeoylquinic acid (**4**), 4,5-dicaffeoylquinic acid (**5**), 3,4-dicaffeoylquinic acid (**6**), chlorogenic acid methyl ester (**7**), cryptochlorogenic acid methyl ester (**8**) and neochlorogenic acid methyl ester (**9**). Chlorogenic acid analogues (**1**–**3**) showed activity against HBV DNA replication with IC_50_ values of 5.5 (SI > 250.1), 13.7 (SI > 115.0) and 7.3 (SI > 249.9) μM; dicaffeoyl analogues (**4**–**6**) significantly inhibited HBV DNA replication with IC_50_ values of 6.4 μM (SI > 256.1), 9.8 μM (SI > 184.8) and 6.1 μM (SI > 184.8), as well as moderate activity against the secretions of HBsAg and HBeAg; the methylated analogues (**7**–**9**) dramatically decreased the activity against HBV DNA replication, with IC_50_ values of 272.3 (SI > 6.2), 175.3 (SI > 11.2) and 144.7 (SI > 20.2) μM, respectively. This result revealed the anti-HBV active constituents of *A. capillaris* and provided valuable information for the potential use of chlorogenic acid analogues as novel non-nucleoside anti-HBV candidates. Furthermore, seven new enynes, 8-(*Z*)-decene-4,6-diyne-1,3,10-triol (**10**), 1,3*S*,8*S*-trihydroxydec-9-en-4,6-yne (**11**), and 3*S*,8*S*-dihydroxydec-9-en-4,6-yne 1-*O*-*β*-d-glucopyranoside (**12**), 8*S*-deca-9-en-4,6-diyne-1,8-diol (**13**), (*S*)-deca-4,6,8-triyne-1,3-diol (**14**), 3*S*-hydroxyundeca-5,7,9-triynoic acid (**15**), 3*S*-hydroxyundeca-5,7,9-triynoic acid 3-*O*-*β*-d-glucopyranoside (**16**), and one new glucosyl caffeoate, 1-*O*-ethyl-6-*O*-caffeoyl-*β*-d-glucopyranose (**17**), together with 34 known compounds were also obtained from this plant. Most of the isolates showed good to moderate activity, and especially, pumilaside A could inhibit both the secretions of HBsAg and HBeAg and HBV DNA replication with IC_50_ values of 15.02 μM (SI = 111.3), 9.00 μM (SI = 185.9) and 12.01 μM (SI = 139.2); protocatechuic acid and caffeic acid exhibited inhibition on HBV DNA replication with IC_50_ values of 32.60 (SI > 255.8) and 16.83 (SI > 255.6) μM; Compound **16** could significantly inhibit the secretions of HBsAg and HBeAg, and HBV DNA replication with IC_50_ values of 197.2 (SI > 5.1), 48.7 (SI > 20.5) and 9.8 (SI > 102) μM [8–11].

In order to characterize the anti-HBV constituents of *A. scoparia*, the bioassay in vitro suggested that the 90% ethanol extract showed inhibition on the secretions of HBsAg and HBeAg with inhibition rates of 36.5 ± 8.1 and 25.0 ± 6.7% (1 mg/mL), and HBV DNA replication with inhibition rate of 49.3 ± 9.7% (0.25 mg/mL). As a result, three new compounds, 4-pyridoneglucoside (**18**), 3*S*,8*S*-dihydroxydec-9-en-4,6-yne 1-*O*-(6′-*O*-caffeoyl)-*β*-d-glucopyranoside (**19**) and 3*S*,8*S*-dihydroxydec-9-en-4,6-yne 1-*O*-(2′-*O*-caffeoyl)-*β*-d-glucopyranoside (**20**) were obtained from the active part under the guidance of LCMS analyses. 4-Pyridoneglucoside (**18**) is an unusual 4-pyridone glucoside that could inhibit the secretions of HBsAg and HBeAg with IC_50_ values of 0.61 mM (SI = 2.1) and 0.88 mM (SI = 1.4), respectively, and HBV DNA replication with an IC_50_ value of 0.47 mM (SI = 2.7). Compound **19** displayed inhibition on HBV DNA replication with an IC_50_ value of 0.07 mM (SI = 23.6), and compound **20** showed slightly decreased activity with an IC_50_ value of 0.12 mM (SI = 17.1) [12].

**Fig. 1:**
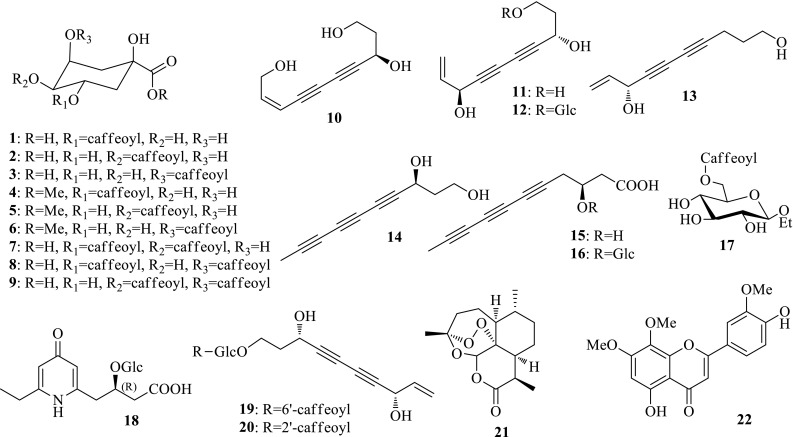
Structures of compounds **1**–**22**

*Artemisia annua* (Huanghuahao) is famous for the production of artemisinin that forms the basis of the most important treatments of malaria. Romero et al. reported that artemisinin (**21**) showed inhibition on HBsAg secretion with an IC_50_ value of 55 μM [13]. 5,4′-Dihydroxyl-6,7,3′-trimethoxyflavone (**22**) from *Artemisiae argyi* (Ai) could suppress the secretions of HBsAg and HBeAg with IC_50_ values of 8.1 and < 3.5 mg/L [14] (Fig. 1).

### *Swertia* Plants

Plants of the genus *Swertia* (Gentianaceae) are annual or perennial herbs, which are mainly distributed in Asia, Africa and North America. Of the total 170 species, 79 species and 10 variants are present in China. There are about 30 *Swertia* plants are used as medicinal herbs in treating hepatitis, cholecystitis, pneumonia, osteomyelitis, dysentery and scabies. The detailed investigation on *Swertia* plants is valuable for searching new anti-HBV candidates.

**Fig. 2:**
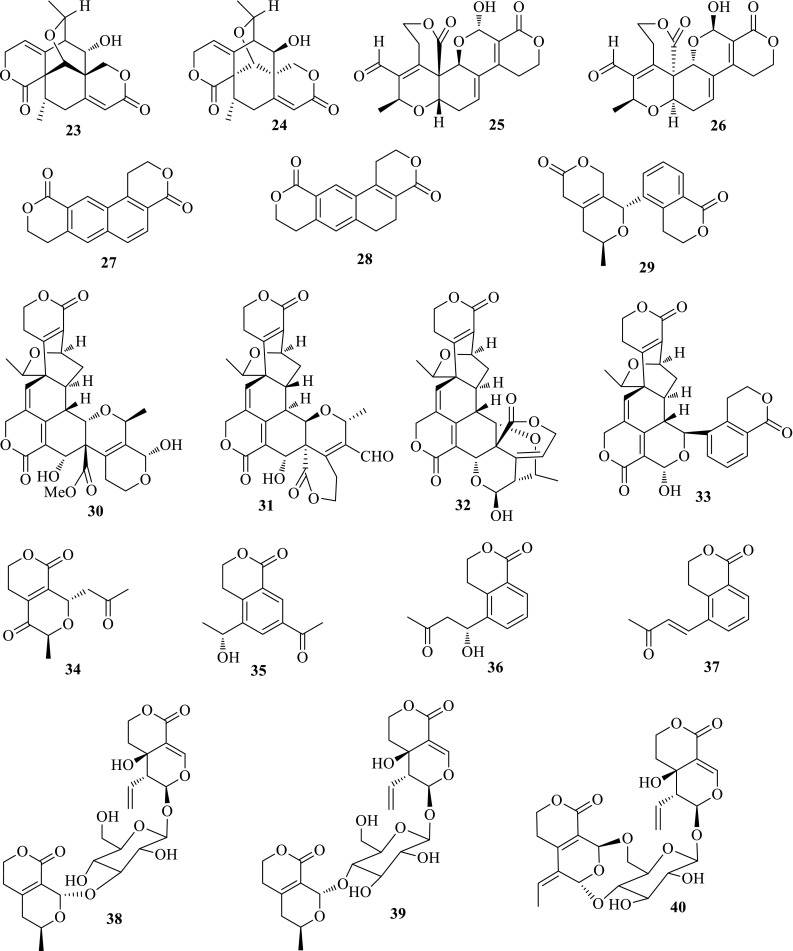
Structures of compounds **23**–**40**

*Swertia mileensis* (Qingyedan) belonging to the *Swertia* genus of the family Gentianaceae is an endemic Chinese herb to Yunnan Province (China). This herb has long been used for acute and chronic hepatitis in the Yi and Hani minority areas of China. *S. mileensis* is the only plant in *Swertia* genus, which is documented in Chinese Pharmacopoeia (1977–2015). Previous investigation revealed the liver protective effects of *S. mileensis* and the active constituents, e.g. sweroside, swertisin, and oleanolic acid, whereas the anti-HBV active constituent is still unclear. The in vitro anti-HBV screening manifested that the ethanol extract of *S. mileensis* was more effective than the water part. The following bioassay-guided isolation gave rise to 47 compounds, involving 29 new and 13 active ones. Swerilactones A and B (**23**, **24**) with an unprecedented 6/6/6/6/6 pentacyclic ring system are the first cases of C_18_-skeleton secoiridoid dimers. Their structures were confirmed by detailed spectroscopic data and X-ray crystallographic analyses. Swerilactone A showed anti-HBV property against HBsAg secretion with an IC_50_ value of 3.66 mM, and against HBeAg secretion with an IC_50_ value of 3.58 mM. However, swerilactone B exhibited no activity at the highest tested concentration of 4.64 mM. Swerilactones C and D (**25**, **26**) with a 6/6/6/6/6 pentacyclic ring system are another type of secoiridoid dimers with C_20_ skeleton, and their structures were determined by X-ray crystallographic analyses. Swerilactones C and D both showed inhibitory activities against the secretions of HBsAg with IC_50_ values of 1.24 and 2.96 mM, and HBeAg with IC_50_ values of 0.77 and 1.47 mM, respectively. Swerilactones E and F (**27**, **28**) are two unusual C_16_-skeleton lactones with a naphthyl or dihydronaphthyl ring, and swerilactone G (**29**) is an unusual C_18_-skeleton *bis*-secoiridoid connected by C–C bond. Swerilactone E showed significant anti-HBV activity against the secretion of HBsAg with an IC_50_ value of 0.22 mM (SI = 9.8) and HBeAg with an IC_50_ value of 0.52 mM (SI = 4.2), which was more potent than its dehydrogenized derivative swerilactone F [IC_50(HBsAg)_ = 0.70 mM, SI = 2.11; IC_50(HBeAg)_ > 6.78 mM, SI < 1]. Swerilactones H–K (**30**–**33**) are four novel C_29_-skeleton secoiridoid trimers, and their structures were determined by extensively spectroscopic and X-ray crystallographic diffraction analyses. Swerilactones H–K exhibited high inhibition on HBV DNA replication with IC_50_ values ranging from 1.53 to 5.34 μM, and less cytotoxicity at the highest tested concentration. Swerilactone I also showed inhibitory activity against the secretions of HBsAg (IC_50_ = 440 μM) and of HBeAg (IC_50_ = 500 μM). From a biogenetic point of view, swerilactones A and B are derived from two molecules of C_9_ secoiridoids; swerilactones C and D are polymerized by two C_10_ secoiridoids; and swerilactones H–K arise from two C_10_ and one C_9_ secoiridoids. Swerilactone L (**34**) is an unusual C_12_-skeleton secoiridoid, and swerilactones M–O (**35–37**) are three new C_13_-skeleton aromatic secoiridoids. The biological origin for the additional C_2_ and C_3_ parts is still unclear. Swerilactones L and M showed moderate activity against the secretions of HBsAg (IC_50_ = 1.47 and 1.20 mM, respectively) and HBeAg (IC_50_ = 0.88 and > 2.69 mM, respectively) [15–19] (Fig. 2).

**Fig. 3:**
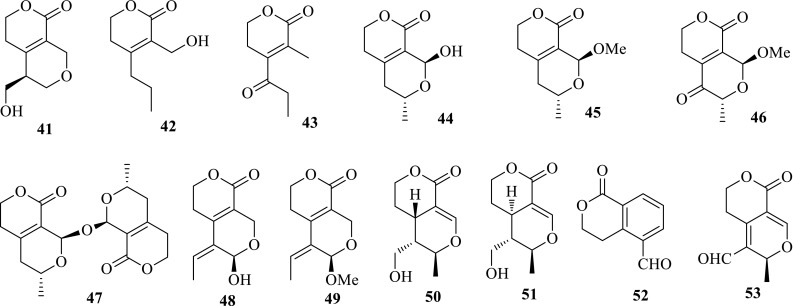
Structures of compounds **41**–**53**

Swerilactosides A–C (**38**–**40**) are three unusual secoiridoid glycoside dimers, which further enriched the skeleton types of secoiridoid glycosides. In addition to the above mentioned compounds, 11 new secoiridoid aglycones, including six C_9_-skeletons (swerimilegenins A–F, **41**–**46**), one *bis*-C_9_-skeleton (swerimilegenin G, **47**), and four C_10_-skeletons (swerimilegenins H–K, **48**–**51**) were further obtained from the low-polarity part of *S. mileensis*, as well as six known ones. Two known compounds, erythrocentaurin (**52**) and gentiogenal (**53**) showed moderate anti-HBV activity on HepG2.2.15 cell line in vitro. Erythrocentaurin could inhibit the HBsAg secretion and HBV DNA replication with IC_50_ values of 1.39 and 0.96 mM; gentiogenal showed activity inhibiting the secretions of HBsAg and HBeAg with IC_50_ values of 3.92 and 2.99 mM, respectively [20, 21] (Fig. 3).

*Swertia leducii* (Mengzi Zhangyacai) is an annual herbaceous plant mainly distributed in Mengzi County of the Yunnan Province. Many traits of *S. leducii* are similar to those of *S. mileensis*, except for petioles and flowers. Due to their close morphology, *S. leducii* was always used as the alternative for *S. mileensis* in producing “Qing-Ye-Dan” medicines. In order to clarify the anti-HBV constituents, a pair of novel enantiomeric lactones, (±)-sweriledugenin A (**54**, **55**), were isolated from *S. leducii* under the guidance of LCMS investigation. Both (+)- and (−)-sweriledugenin A showed activities inhibiting HBV DNA replication with IC_50_ values of 36.86 (SI = 10.5) and 26.55 (SI = 31.6) μM on the HepG 2.2.15 cell line in vitro [22]. This investigation is a valuable attempt for guided isolation from a completed natural complex.

*Swertia punicea* (Zihong Zhangyacai), the congener species of *S. mileensis*, are used for the treatment of hepatitis in the folk of Yunnan Province. The anti-HBV screening manifested that the EtOAc extract of *S. punicea* showed inhibitory activities on HBsAg and HBeAg with IC_50_ values of 0.69 and 0.15 mg/mL, respectively. In order to clarify the active components, swerpunilactone A (**56**) and its plausible precursors, (±)-gentiolactone (**58**) and bellidifolin (**59**) were isolated from the whole plants of *S. punicea*. Simultaneously, swerpunilactone B (**57**), (±)-gentiolactone, and norbellidifolin (**60**) were further obtained from *S. hispidicalyx* and *S. yunnanensis*. Swerpunilactones A and B as two unusual xanthone and secoiridoid heterodimers showed similar activity inhibiting HBsAg secretion with IC_50_ values of 0.25 (SI = 1.2) and 0.29 (SI > 1.4) mM, and HBV DNA replication with IC_50_ values of 0.18 (SI > 1.7) and 0.19 (SI > 2.2) mM. Bellidifolin could obviously inhibit HBV DNA replication with an IC_50_ value of 0.09 (SI > 10.9) mM. Norbellidifolin showed inhibition on both HBsAg and HBeAg secretions and HBV DNA replication with IC_50_ values of 0.77 (SI > 6.2), < 0.62 (SI > 7.8) and 0.58 (SI > 8.3) mM, respectively [23]. This is the first report of xanthone and secoiridoid heterodimers connected with C–C bond.

**Fig. 4:**
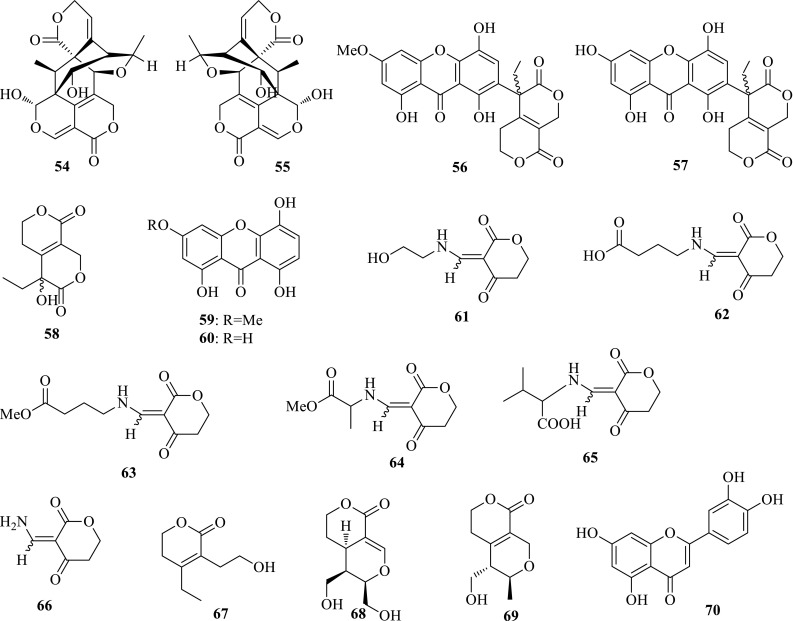
Structures of compounds **54**–**70**

*Swertia macrosperma* (Dazi Zhangyacai), the congeneric species of *S. mileensis*, is widely used for curing hepatitis in Yunnan Province, China. Previous phytochemical studies demonstrated that xanthones, triterpenoids, and secoiridoid glycosides were the main constituents, whereas their anti-HBV effects remain unclear. The bioassay on HepG2.2.15 cell line in vitro revealed that the water extraction of *S. macrosperma* showed activity inhibiting HBsAg and HBeAg with IC_50_ values of 0.22 (SI = 19.0) and 0.14 (SI > 30.0) mg/mL. Subsequent separation on this part provided eight new and 46 known compounds, seven of which showed anti-HBV potency. Gentiocrucines A–E (**61**–**65**) as five unusual lactonic enamino ketones (LEKs) are the only cases after the first report of gentiocrucine (**66**). Compared to gentiocrucine, gentiocrucines A–E maintain prolific substituents on N-7, and their isolation further enriches the structure types of LEKs. In addition, gentiocrucines A–E and gentiocrucine were also obtained from the congener plant, *S. angustifolia*. Gentiocrucine A (**61**) showed moderate activity inhibiting the secretions of HBsAg and HBeAg with IC_50_ values of 3.14 (SI > 1.2) and 3.35 (SI > 1.2) mM. Swermacrolactones A–C (**67**–**69**) as three new secoiridoid aglycones showed no activity and cytotoxicity at the tested concentrations. One known flavonoid, luteolin (**70**) exhibited inhibitory activity on the secretions of HBsAg and HBeAg with the same IC_50_ value of 0.02 mM (SI = 4.0) [24–26] (Fig. 4).

*Swertia kouitchensis* (Guizhou Zhangyacai), a perennial herb mainly distributed in Southern China, is widely used as a fork medicine to treat jaundice, indigestion and sore throat. Detailed investigation on the ethanol part of *S. kouitchensis* yielded three new secoiridoids, swertiakoulactone (**71**) and swertiakosides A and B (**72**, **73**), as well as 28 known compounds. Swertiakoside B is a new secoiridoid glycoside in which the glycosyl is attached to the aglycon by the OH-1′ and OH-2′ simultaneously. Swertiakoulactone showed moderate activities inhibiting the HBsAg secretion (IC_50_ = 1.10 mM, SI = 4.4) and HBV DNA replication (IC_50_ = 1.16 mM, SI = 4.1) [27, 28].

*Swertia cincta* (Xinan Zhangyacai), the congener plant of *S. mileensis*, is generally used to treat hepatitis in the folk region of Yunnan Province (China). The in vitro anti-HBV bioassay manifested that the 90% ethanol extract of *S. cincta* could inhibit the secretions of HBsAg and HBeAg, and HBV DNA replication with IC_50_ values of 151.5 μg/mL (SI > 20.0), 53.7 μg/mL (SI > 40.8) and 121.9 μg/mL (SI > 24.0), respectively. In order to clarify its active constituents, extensive investigation on the ethanol extract of *S. cincta* yielded six new compounds, including five secoiridoid glycosides, swericinctosides A and B (**74**–**75**), 9-*epi*-swertiamarin (**76**), 2′-*O*-mhydroxybenzoyl swertiamarin (**77**), 4″-*O*-acetyl swertianoside E (**78**), and one unusual lactone enol ketone, 3-(hydroxymethyl ene) dihydro-2*H*-pyran-2,4(3*H*)-dione (**79**), as well as 18 known ones. The most active swertiaside (**80**) exhibited activity against HBV DNA replication with an IC_50_ value of 0.05 mM (SI = 29.1), as well as moderate activity against HBsAg secretion (IC_50_ = 0.79 mM, SI = 2.0). Swericinctosides A and 9-*epi*-swertiamarin also showed moderate activity inhibiting HBsAg secretion with IC_50_ values of 0.32 and 1.06 mM, and HBV DNA with IC_50_ values of 0.46 and 0.62 mM, respectively [29, 30].

*Swertia angustifolia* (Xiaye Zhangyacai), the congener plant of *S. mileensis*, is used for treating hepatitis and cholecystitis in the folk region of Yunnan Province (China). The phytochemical investigation on this plant gave rise to seven new secoiridoids, swertianglide (**81**) and swertianosides A–F (**82**–**87**), together with 29 known compounds. Swertianoside A, an unusual secoiridoid glycoside dimer with two molecules of secoiridoids connected with a glucosyl group, showed significant activities inhibiting the secretions of HBsAg (IC_50_ = 0.18 mM, SI = 3.1) and HBeAg (IC_50_ = 0.12 mM, SI = 4.7), and HBV DNA replication (IC_50_ = 0.22 mM, SI = 2.5). As a comparison, other secoiridoid glycosides displayed no activity or cytotoxicity at the tested concentration. This result suggests the importance of two secoiridoid aglycones in the structure [31, 32].

**Fig. 5:**
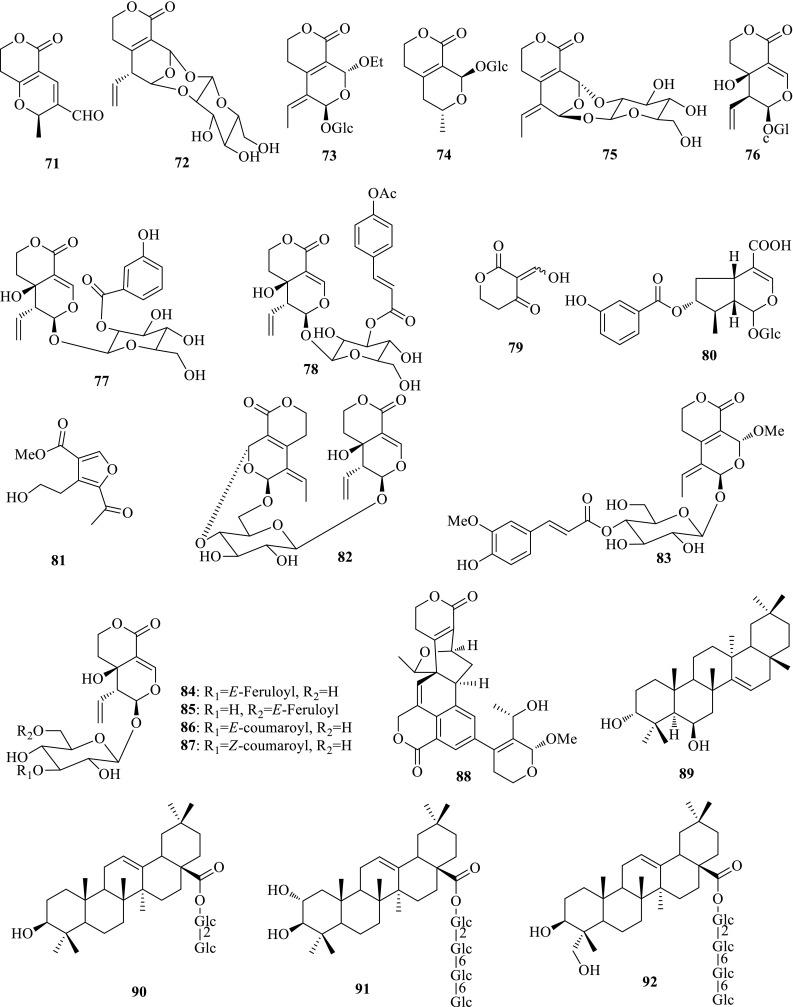
Structures of compounds **71**–**92**

*Swertia yunnanensis* (Yunnan Zhangyacai) as the congener plant of *S. mileensis* is always used as the alternative of Qingyedan for treating jaundice, hepatitis, and cholecystitis in Yunnan, Sichuan, and Guizhou Provinces of China. The in vitro anti-HBV bioassay manifested that the ethanol extracts of *S. yunnanensis* showed significant inhibition on the secretions of HBsAg and HBeAg with IC_50_ values of 0.79 mg/mL (SI = 2.4) and 0.34 mg/mL (SI = 5.4), respectively, and on HBV DNA replication with an IC_50_ value of 0.08 mg/mL (SI = 18.9). Detailed investigation on the ethanol part yielded 24 compounds, involving five new ones (**88**–**92**). Sweriyunnanlactone A (**88**) with a phenyl ring is the first example of C_28_-skeleton secoiridoid trimer which was obtained under the guidance of LC–MS analysis. Its structure was determined by extensive HRESIMS, 1D and 2D NMR spectroscopic data, and GIAO ^13^C NMR calculation. Sweriyunnanlactone A showed inhibition on HBV DNA replication with an IC_50_ value of 60.76 μM (SI = 12.6) on HepG 2.2.15 cell line in vitro. Sweriyunnangenin A (**89**), sweriyunnanosides A (**90**), B (**91**) and C (**92**) are four new oleanane-type triterpenes with no or weak anti-HBV activity. Two xanthones, 1,8-dihydroxy-3,5-dimethoxyxanthone and norswertianolin displayed remarkable inhibition on HBV DNA replication with IC_50_ values of 0.07 (SI > 71.4) and 0.01 (SI > 269.0) mM, respectively. Neolancerin showed anti-HBV activity inhibiting the secretions of HBsAg (IC_50_ = 0.21 mM, SI = 9.7) and HBeAg (IC_50_ = 0.04 mM, SI = 51.0), and HBV DNA replication (IC_50_ = 0.09 mM, SI = 22.7) [33, 34] (Fig. 5).

*Swertia mussotii* (Chuanxi Zhangyacai) is a well-known Tibetan folk medicine (Zangyinchen) to treat hepatitis. According to the preliminary bioassay in vitro, the ethanol extract of *S. mussotii* showed significant inhibitory activity on the secretions of HBsAg and HBeAg with IC_50_ values of 0.55 mg/mL (SI = 2.1) and 0.19 mg/mL (SI = 6.2), respectively, and HBV DNA replication with an IC_50_ value of 0.043 mg/mL (SI = 27.2). Subsequent investigation on the active part gave rise to 69 compounds, involving two new xanthones, 8-*O*-*β*-d-glucopyranosyl-1-hydroxy-2,3,5-trimethoxyxanthone (**93**) and 8-*O*-[*β*-d-xylopyranosyl-(1 → 6)-*β*-d-glucopyranosyl]-1-hydroxy-2,3,5-trimethoxyxanthone (**94**), and seven new secoiridoids, (−)-swermusic acids A (**95**) and B (**96**), and (−)-swerimuslatone A (**97**), four new secoiridoid glycosides, 6′-*O*-formylsweroside (**98**), 6′-*O*-formylgentiopicroside (**99**), 6′-*O*-acetylamarogentin (**100**) and 6′-*O*-acetylamaronitidin (**101**), and two new linear monoterpenes, swerimusic acids C (**102**) and D (**103**). The structures of the new compounds were elucidated on the basis of extensive spectroscopic analyses. Eight xanthones exhibited significant activity inhibiting HBV DNA replication with IC_50_ values from 0.01 to 0.13 mM. Especially, mangiferin and isoorientin could inhibit HBV DNA replication with IC_50_ values of 0.01 (SI > 145.2) and 0.02 (SI > 177.0) mM, respectively. This is the first time to systematically investigate the xanthones and their anti-HBV activity in *Swertia* plants. Ethyl 3,4-dihydroxybenzoate and ethyl 2,5-dihydroxybenzoate also showed inhibition on HBsAg secretion with IC_50_ values of 0.14 (SI > 41.6) and 0.23 (SI > 32.3) mM; 2-C-*β*-d-glucopyranosyl-1,3,7-trihydroxyxanthone could suppress HBeAg secretion with an IC_50_ value of 0.04 mM (SI = 47.8) [35–37].

*Swertia patens* (Xiejing Zhangyacai), mainly distributed in Yunnan and Sichuan provinces of China, is used as a folk medicine to treat hepatitis in folk. However, its chemical constituents and bioactivities were seldom reported. In continuing efforts to discover anti-HBV active compounds from *Swertia*, phytochemical investigation on *S*. *patens* led to the isolation of two new secoiridoids, swerpatic acid (**104**) and swerpalactone (**105**), together with 28 known compounds. Swerpatic acid (**106**) is an unusual C_8_-skeleton secoiridoid aglycone. Two known compounds, (+)-dehydrodiconiferyl alcohol and dehydrozingerone showed moderate activities inhibiting the secretion of HBsAg with IC_50_ values of 1.94 mM (SI = 1.1) and 0.50 mM (SI = 2.9), while other compounds exhibited obvious cytotoxicity on Hep G 2.2.15 cell with CC_50_ values from 0.24 to 2.06 mM [38, 39].

*Swertia chirayita* (Yindu Zhangyacai) is distributed throughout the temperate Himalaya area. The whole plant is widely used by local people for the treatment of hepatitis, inflammation and digestive diseases. Based on the preliminary bioassay in vitro, the EtOH extract showed inhibition on the secretions of HBsAg and HBeAg with IC_50_ values of 1.08 mg/mL (SI > 2.0) and 0.11 mg/mL (SI > 19.6), and HBV DNA replication with an IC_50_ value of 0.17 mg/mL (SI = 12.9). Bioassay-guided fractionation of the active part resulted in four new compounds, swertiachiralatone A (**89**), swertiachoside A (**107**), swertiachirdiol A (**108**) and swertiachoside B (**109**), together with 26 known ones. All the isolates were evaluated for their anti-HBV activity on HepG 2.2.15 cells in vitro. Four compounds showed activity inhibiting HBsAg secretion; two compounds showed activity inhibiting HBeAg secretion; and nine compounds exhibited activity inhibiting HBV DNA replication. In particular, 1-hydroxy-3, 7-dimethoxyxanthone (**110**) displayed obviously inhibition on HBV DNA replication with an IC_50_ value of 0.16 mM (SI > 12.4). (+)-Cycloolivil-4′-*O*-*β*-d-glucopyranoside (**111**) exhibited inhibiting on the secretions of HBsAg and HBeAg with IC_50_ values of 0.31 mM (SI = 4.3) and 0.77 mM (SI = 1.8), and HBV DNA replication with an IC_50_ value of 0.29 mM (SI = 4.7) [40]. This work provides valuable information for understanding the anti-HBV active constituents of *S. chirayita*.

*Swertia hispidicalyx* (Maoe Zhangyacai) is mainly distributed in Tibet of China and Nepal. The first study on the ethanol part gave rise to 11 compounds, two of which showed anti-HBV activity. In particular, 1,5,8-trihydroxy-3-methoxyxanthone (**112**) showed potent activity against HBeAg secretion with an IC_50_ value of 0.35 mM (SI > 2.8) and HBV DNA replication with an IC_50_ value of 0.09 mM (SI > 10.9) [41].

*Swertia delavayi* (Lijiang Zhangyacai) known as “Gan-Yan-Cao” or “Zou-Dan-Cao” is distributed in northwestern Yunnan and southern Sichuan of China. This plant is widely used as a fork medicine for treating jaundice, hepatitis and cholecystitis in Tibetan and Naxi medicines. According to the preliminary bioassay in vitro, the ethanol extracts of *S. delavayi* exhibited significant inhibition on the secretions of HBsAg and HBeAg with IC_50_ values of 1.64 mg/mL (SI = 2.3) and 1.13 mg/mL (SI = 3.6), and HBV DNA replication with an IC_50_ value of 0.34 mg/mL (SI > 7.3). As a continuous search for anti-HBV active constituents from natural sources, 15 compounds were obtained from the active part of this plant. Especially, isovitexin (**113**) exhibited significant activity inhibiting HBV DNA replication with an IC_50_ value of 0.05 (SI = 19.8) mM, and the secretion of HBeAg with an IC_50_ values of 0.23 (SI = 4.3) mM [42].

**Fig. 6:**
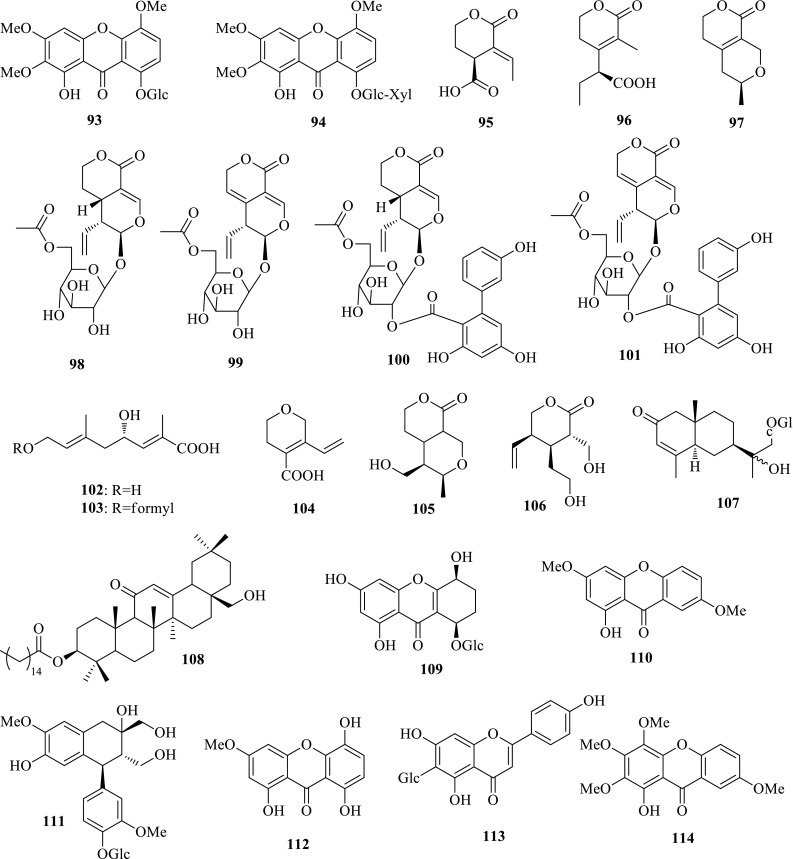
Structures of compounds **93**–**114**

In addition to the *Swertia* plants mentioned above, *Halenia elliptica* (Tuoyuanye Huamao) that is also used as the substituent of Qing-Ye-Dan for treating hepatitis by the local people was further investigated. *H. elliptica* (Tuoyuanye Huamao) within the Gentianaceae family is an annual herb widely distributed in Southwest, Northwest and North China. The bioassay in vitro suggested that the ethanol part of this plant showed inhibition on HBsAg and HBeAg secretions with IC_50_ values of 1.07 (SI > 4.0) and 1.39 (SI > 3.1) mg/mL. Subsequent isolation on ethanol part generated 17 compounds, four of which showed anti-HBV activity. In consist with the previous report, norbellidifolin was revealed with activiy inhibiting the secretions of HBsAg and HBeAg and HBV DNA replication. The known xanthone, 1-hydroxy-2,3,4,7-tetramethoxyxanthone (**114**) also showed activity inhibiting HBsAg and HbeAg secretions with IC_50_ values of 2.50 (SI > 2.2) and 3.61 (SI > 1.5) mM, respectively [43, 44] (Fig. 6).

### Other Plants

*Alisma orientalis* (Zexie) is widely cultivated in China and Japan, and its dried rhizomes are famous crude drugs for the treatment of diabetes and diuretics. In order to characterize the anti-HBV active constituents, detailed investigation on this plant yielded two new sesquiterpenes, alismorientols A (**115**) and B (**116**), one new protostane-type triterpene, alisol O (**117**), and seven known ones, alisol A (**118**), alisol A 24-acetate (**119**), 25-anhydroalisol A (**120**), 13*β*,17*β*-epoxyalisol A (**121**), alisol B 23-acetate (**122**), alisol F (**123**) and alisol F 24-acetate (**124**). Anti-HBV bioassay on HepG 2.2.15 cell line in vitro revealed that alismorientol A exhibited inhibition on the secretions of HBsAg and HBeAg, with IC_50_ values of 1.1 (SI = 16.7) and 14.7 (SI > 1.2) μM, respectively. Seven protostane-type triterpenes (**118**–**124**) showed potent anti-HBV activity, and especially, alisol F 24-acetate could inhibited the hepatitis B surface antigen (HBsAg) and hepatitis B e antigen (HBeAg) secretions with IC_50_ values of 7.7 (SI = 18.5) and 5.1 (SI = 28.0) μM [45, 46]. This is the first time to reveal the anti-HBV active constituents of *A. orientalis*.

**Fig. 7:**
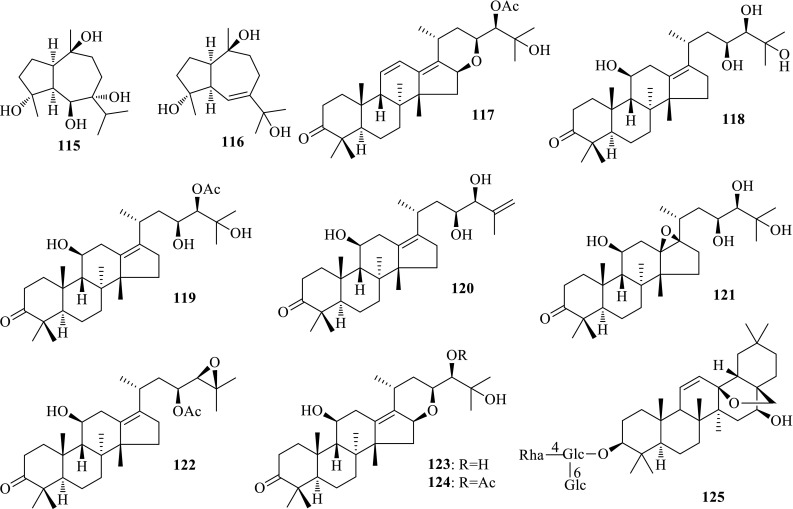
Structures of compounds **115**–**125**

Saikosaponins are the major components of *Bupleurum* spp. with immunomodulatory, hepatoprotective, antitumor and antiviral activities. Lin et al. reported saikosaponin C (**125**) with potent activity inhibiting HBsAg (IC_50_ = 11 μM) and HBV DNA (IC_50_ = 13.4 μM) [47] (Fig. 7).

**Fig. 8:**
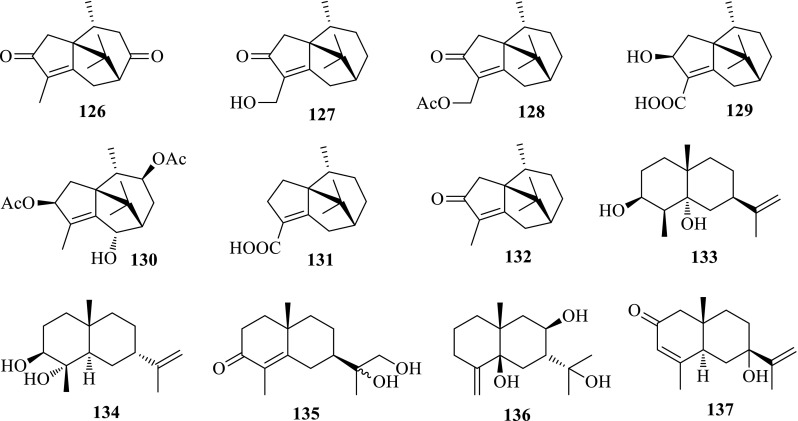
Structures of compounds **126**–**137**

*Cyperus rotundus* Linneus (purple nutsedge), belonging to the family Cyperaceae, is a perennial herb indigenous to China and distributed in tropical and subtropical regions throughout the world. The rhizomes of *C. rotundus* (Xiangfu) recorded in every edition of Chinese Pharmacopoeia have been widely used as a traditional Chinese medicine to cure liver diseases, pain and women’s diseases. The bioassay in vitro suggested the ethanol extraction showed anti-HBV activity inhibiting HBsAg and HBeAg secretions with IC_50_ values of 462.4 (SI = 1.3) and 42.8 (SI = 14.1) mg/mL, and HBV DNA replication with an IC_50_ value of 29.0 (SI = 20.8) mg/mL. Bioassay-guided separation gave rise to the active Frs. B–D, from which five new patchoulane-type sesquiterpenoids, cyperene-3,8-dione (**126**), 14-hydroxycyperotundone (**127**), 14-acetoxycyperotundone (**128**), 3*β*-hydroxycyperenoicacid (**129**) and sugetriol-3,9-diacetate (**130**), along with 32 known ones were obtained. The eudesmane-type sesquiterpenoids, 10-*epi*-eudesm-11-ene-3*β*,5*α*-diol (**133**), 3*β*,4*α*-dihydroxy-7-*epi*-eudesm-11(13)-ene (**134**), 7*α*(*H*),10*β*-eudesm-4-en-3-one-11,12-diol (**135**) and rhombitriol (**136**) significantly inhibited the HBV DNA replication with IC_50_ values of 13.2, 13.8, 19.7 and 11.9 μM, with high SI values of 250.4, 125.5, > 259.6 and 127.5, respectively. Two patchoulane-type sesquiterpenoids, 3*β*-hydroxycyperenoicacid (**129**) and cyperenoicacid (**131**) effectively suppressed the secretion of HBsAg in a dose-dependent manner with IC_50_ values of 46.6 (SI = 31.0) and 77.2 (SI = 1.7) μM, respectively. 14-Hydroxycyperotundone (**127**), cyperotundone (**132**) and 7-*epi*-teucrenone (**137**) possessed moderate activities against HBeAg secretion with IC_50_ values of 162.5 (SI = 13.3), 399.2 (SI = 10.6) and 285.3 (SI = 15.5) μM. This is the first study to reveal the anti-HBV constituents of *C. rotundus*, suggesting that the eudesmane-type sesquiterpenoids might contribute to the anti-HBV activity of Xiangfu [48] (Fig. 8).

**Fig. 9:**
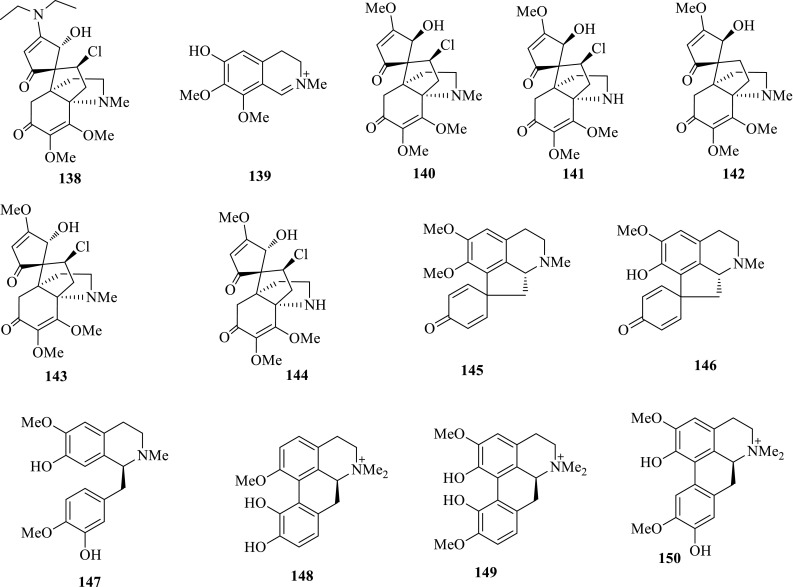
Structures of compounds **138**–**150**

*Hypserpa nitida* (Yehuateng), mainly distributed in China and south Asia, is a medicinal herb for treating inflammation in the folk. The 90% EtOH extract of this plant were first revealed with anti-HBV potency inhibiting HBsAg and HBeAg secretions with IC_50_ values of 0.44 and 0.92 mg/mL. Following investigation gave rise to two new alkaloids, hypserpanines A and B (**138**, **148**) and 11 known compounds, phenolbetain (**139**), acutumine (**140**), acutumidine (**141**), dechloroacutumine (**142**), dauricumine (**143**), dauricumidine (**144**), pronuciferine (**145**), glaziovine (**146**), *S*-reticuline (**147**), magnoflorine (**149**) and laurifoline (**150**). According to the assay on Hep G2.2.15 cell line in vitro, the acutumine-type alkaloids, hyperpanine A (**138**), acutumidine (**141**), dauricumine (**143**) and dauricumidine (**144**) showed moderate activity, especially dauricumidine (**144**) could inhibit the secretion of HBsAg with an IC_50_ value of 0.450 mM (SI = 4.1); the proaporphine alkaloids, pronuciferine (**145**) and glaziovine (**146**) showed inhibition on HBsAg secretion with IC_50_ values of 0.042 and 0.008 mM, but with obvious cytotoxicity; the aporphine alkaloids **148**–**150** were inactive to both the secretions of HBsAg and HBeAg [49] (Fig. 9).

*Illicium henryi* (Honghuixiang) is a shrub distributed in the southwestern part of China, and its bark and roots have been used as a folk-medicinal herb for dispelling wind-evil and assuaging pain. In order to characterized the active constituents, detailed investigation on the stems and roots of *I. henryi* yielded 39 compounds, involving 7 new ones. The structures of the new compounds were elucidated as dihydrodehydrodiconiferyl alcohol 9-*O*-*β*-*d*-(3″-*O*-acetyl)-xylopyranoside (**151**) and *threo*-4,9,9′-trihydroxy-3,3′-dimethoxy-8-*O*-4′-neolignan 7-*O*-*α*-rhamnopyranoside (**152**) and henrylactones A–E (**153**–**157**) by extensive spectroscopic analyses and X-ray diffraction experiment. Most of the new lignans displayed anti-HBV property, especially (−)-dihydrodehydrodiconiferyl alcohol (**158**) showed moderate activity inhibiting both HBsAg and HBeAg secretions with IC_50_ values of 0.06 (SI = 8.8) and 0.53 (SI = 1.1) mM, respectively. For the sesquiterpenes, tashironin A (**159**) showed an IC_50_ value of 0.48 mM (SI = 6.3) in inhibiting HBsAg secretion, and 0.15 mM (SI = 20.1) in inhibiting HBeAg secretion. Henrylactones A–E (**153**–**157**) also showed moderate activity inhibiting HBsAg secretion with IC_50_ values ranging from 0.098 to 1.85 mM, and HBeAg with IC_50_ values ranging from 0.24 to 2.91 mM [50–52].

**Fig. 10:**
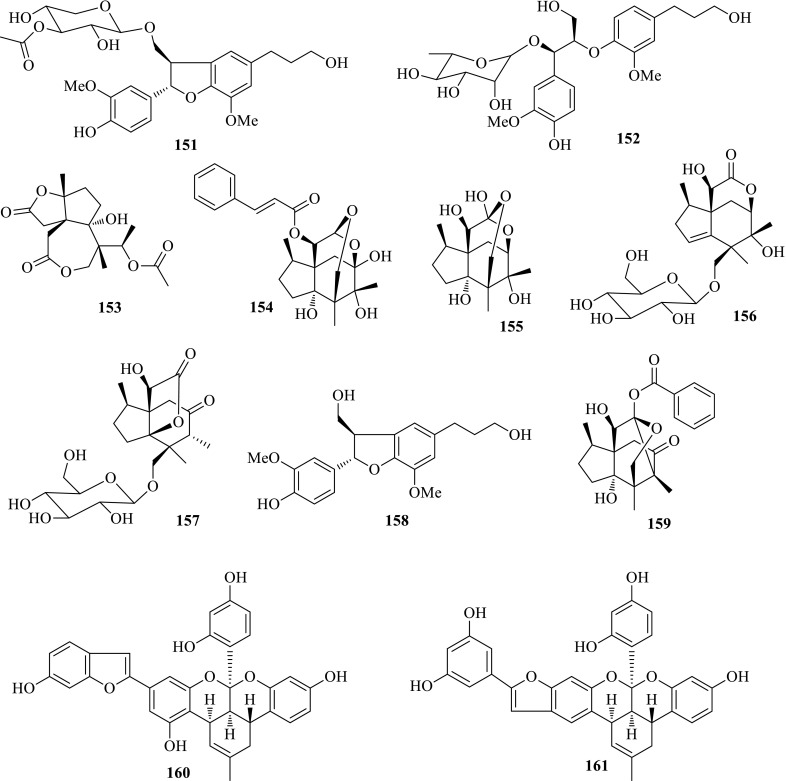
Structures of compounds **151**–**161**

*Morus alba* (Sang) belonging to the genus of *Morus* in Moraceae family is widely cultivated in China, whose leaves are indispensable food for silkworms. Its root bark (Mori cortex, Sang-Bai-Pi) is a traditional Chinese herb recorded in Chinese Pharmacopoeia (2000 edition) for anti-inflammation, antihypertension, hypoglycemic and diuretic purposes. In order to reveal the anti-HBV active constituents, phytochemical investigation on the root bark of *M. alba* provided two polyphenols, mulberrofuran G (**160**) and isomulberrofuran G (**161**), one new flavonoid, sanggenol P (**162**) and nine known compounds. Mulberrofuran G (**160**) exhibited moderate activity against HBV DNA replication, with an IC_50_ value of 3.99 μM (SI = 2.0), but weak activity against HBsAg and HBeAg with SI values less than 1. Isomulberrofuran G (**161**) as the isomer of mulberrofuran G was inactive at the tested concentration. The MS/MS fragmentation pathway for mulberrofuran G and isomulberrofuran G was investigated for the first time, and he characteristic ions at *m/z* 451 and 439 in MS^2^ could be considered as their respective diagnostic ions [53, 54] (Fig. 10).

*Pericampylus glaucus* (Xiyuanteng), a climbing shrub, is widely distributed in southwest China, whose roots are used for treating laryngitis, cough and pulmonary disease, and leaves are applied for fractures and boar bites. Detailed investigation on the ethanolic extract of *P. glaucus* yielded four new alkaloids, periglaucines A–D (**162**–**165**), along with three known ones, norruffscine (**166**), (−)-8-oxotetrahydropalmatine (**167**) and (−)-8-oxocanadine (**168**). Compounds **162**–**167** showed anti-HBV activity on HepG2.2.15 cell line in vitro. The most potent compound **167** could inhibit HBsAg secretion with an IC_50_ value of 0.14 mM (SI = 22.4) [55].

**Fig. 11:**
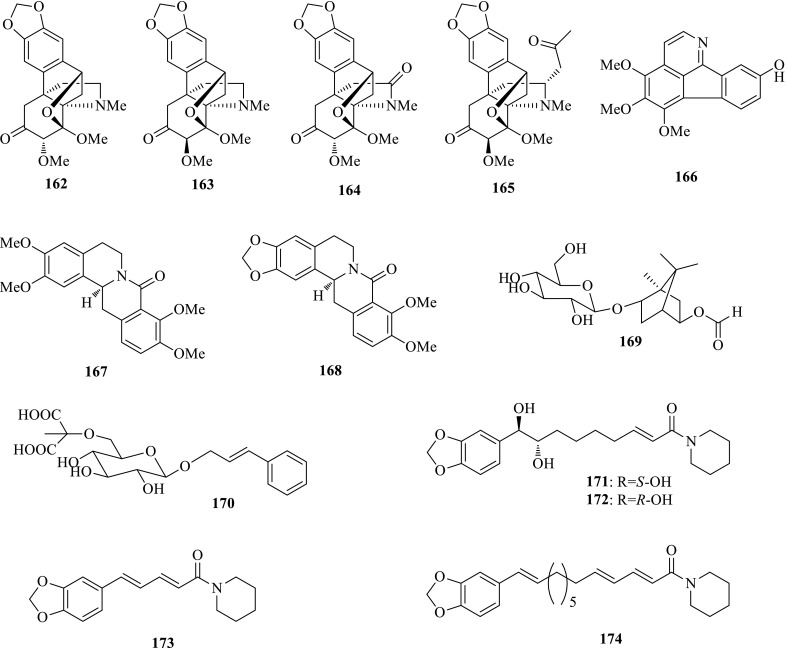
Structures of compounds **162**–**174**

*Piper longum* (Bibo) is a slender climber widely distributed in the tropical and subtropical regions of the world. Its fruits are used for the treatment of anodyne and stomach disease in China. The previous bioassay suggested the ethanol extract of *P. longum* showed anti-HBV activity, from which four new compounds, named longumosides A (**169**) and B (**170**), *erythro*-1-[1-oxo-9(3,4-methylenedioxyphenyl)-8,9-dihydroxy-2*E*-nonenyl]-piperidine (**171**), and *threo*-1-[1-oxo-9(3,4-methylenedioxyphenyl)-8,9-dihydroxy-2E-nonenyl]-piperidine (**172**) were isolated, as well as 30 known ones. Compounds **171** and **172**, piperine (**173**) and guineesine (**174**) possessed inhibitory activity suppressing the secretions of HBsAg and HBeAg with IC_50_ values of 0.13, 0.11, 0.15 and 0.05 mM for HBsAg, and 0.16, 0.11 0.14 and 0.05 mM for HBeAg, respectively. Primary SARs study suggested that a long carbon chain (n > 8) in the amide alkaloids is helpful for the anti-HBV ability [56–58] (Fig. 11).

**Fig. 12:**
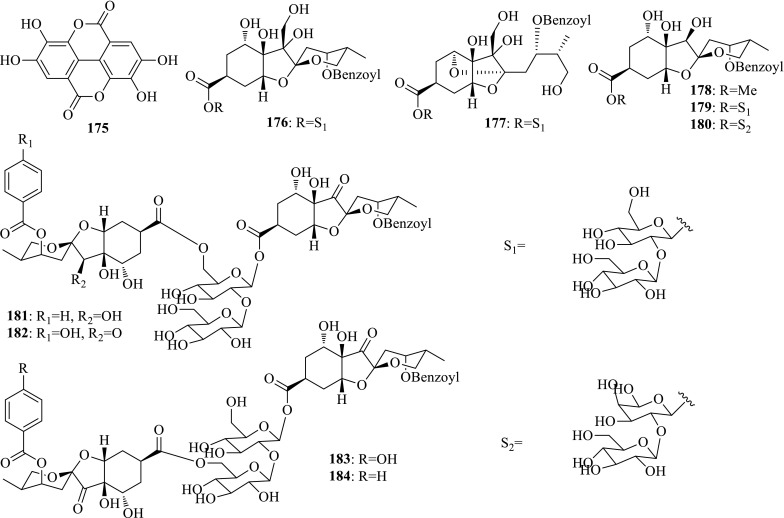
Structures of compounds **175**–**184**

*Phyllanthus urinaria* (Yexiazhu) distributed in most part of China is always used for antiinflammatory, antiviral, antibacterial, and antihepatotoxic purposes. The flavonoid, ellagic acid (**175**), showed potent anti-HBV activity with unique mechanism by targeting HBeAg secretion (IC_50_ = 0.07 μg/mL) [59]. Zhang et al. reported eight new highly oxygenated bisabolane sesquiterpenoid glycosides, phyllaemblicins G1–G8 (**176**–**183**) from the congener plant *Phyllanthus emblica* (Yuganzi). Phyllaemblicins G6–G8 and F (**184**) displayed potential anti-HBV activity, and especially, phyllaemblicin G6 could inhibit HBsAg and HBeAg with IC_50_ values of 8.53 and 5.68 μM [60] (Fig. 12).

*Radix Astragali* (Huangqi), one of the most widely prescribed Chinese medicines, is the dried root of *Astragalus membranaceus* (FISCH.) Bge. var. *mongholicus* (Bge.) Hsiao or *A. membranaceus* (Fisch.) Bge. The major saponin astragaloside IV (**185**) effectively suppressed the secretions of HBsAg and HBeAg with inhibition rates of 23.6 and 22.9% at 100 mg/mL. Furthermore, astragaloside IV showed 64.0% inhibition to serum DHBVs at 120 mg/kg [61].

*Rheum palmatum* (Zhangye Dahuang) is a famous traditional Chinese herb with antibacterial, anticancer and antiviral properties. Li et al. reported two anthraquinones, emodin (**186**) and rhein (**187**) showing inhibition on HBV DNA with an IC_50_ value of 10.48 and 9.89 μg/mL [62].

*Saniculiphyllum guangxiense* (Biandouyecao) the only species of the genus *Saniculiphyllum* (Saxifragaceae), is a perennial herb mainly distributed in Yunnan and Guangxi provinces of China. The first phytochemical investigation resulted in the isolation of two new triterpenoids, 16*β*-hydroxybryodulcosigenin (**188**) and 3*α*-*O*-feruloylolean-12-en-27-oic acid (**189**), together with six known compounds. The nitrile glucoside, menisdaurin (**190**), showed potent activity inhibiting HBV DNA replication with an IC_50_ value of 0.32 mM (SI > 12.0) [63, 64].

*Scutellaria baicalensis* (Huangqin) is a widely used Chinese herbal medicine in antiinflammatory and anticancer therapy. The main constituent, wogonin (**191**), from this plant showed potent anti-HBV activity both in vitro and in vivo. Wogonin could inhibit HBsAg and HBeAg with an IC_50_ value of 4 μg/mL, and DHBV DNA polymerase with an IC_50_ value of 0.57 μg/mL [65].

*Sophora flavescens* (Kushen) widely distributed throughout China is commonly used the treatment of skin diseases and gynecological diseases. Quinolizidine alkaloids are the characteristic constituents in this plant from which Chen et al. reported one new, (+)-12*α*-hydroxysophocarpine, and ten known quinolizidine alkaloids. (+)-Oxysophocarpine (**192**), (−)-sophocarpine (**193**), (+)-lehmannine (**194**), (−)-13,14-dehydrosophoridine (**195**) could significantly inhibited HBsAg secretion by more than 48.3% and HBeAg secretion by 24.6–34.6% at the noncytotoxic concentration of 0.2 lmol/mL. The preliminary SARs suggest the importance of double bond in ring D [66].

**Fig. 13:**
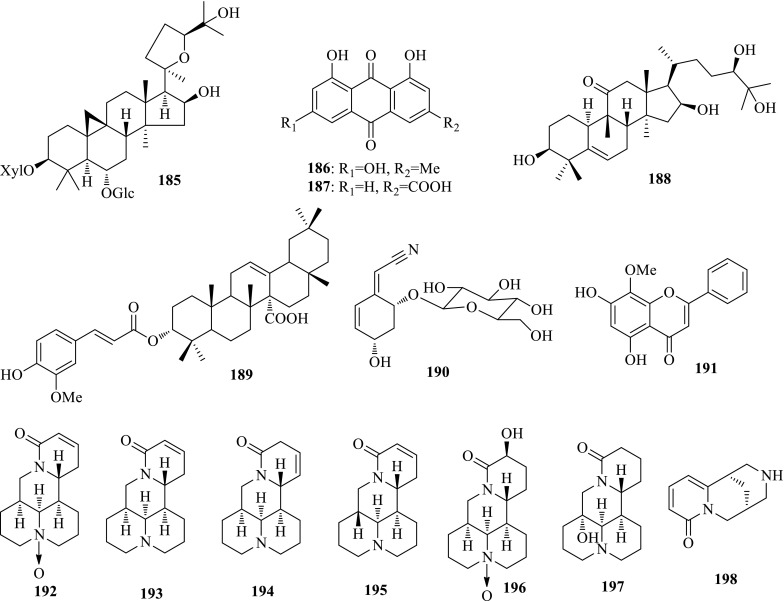
Structures of compounds **185**–**198**

*Sophora tonkinensis* (Yuenanhuai) is mainly present in Guangxi, Guizhou and Yunnan Provinces of China, whose roots and rhizomes are commonly used as the traditional Chinese medicine “Shandougen” for treating acute pharyngolaryngeal infections and sore throat. As a continuous research, Chen et al. further reported one new matrine-type alkaloid, (−)-14*β*-hydroxyoxymatrine (**196**) and five known ones. (−)-14*β*-Hydroxyoxymatrine (**196**), (+)-sophoranol (**197**) and (−)-cytisine (**198**) showed activity against HBsAg secretion with an inhibitory potency of 22.6, 31.1 and 33.2%, and HBeAg secretion with an inhibitory potency of 30.4, 26.3 and 27.8%, respectively [67] (Fig. 13).

## Conclusion and Perspective

This paper summarized the progress of anti-HBV constituents from medicinal plants in China. In total, 198 compounds involving monoterpenes, sesquiterpenes, triterpenes secoiridoids and their polymers, alkaloids, xanthones, lignans, enynes, amides and polyphenols were reviewed. Especially, four secoiridoid trimers, swerilactones H–K can obviously suppress HBV DNA replication with IC_50_ values ranging from 1.53 to 5.34 μM, comparable to the positive drug lamivudine (3TC). With the consideration of the novel structures, they should have different action mechanism with nucleosides. Oxymatrine has been used to treat hepatitis B in clinic with confirmed efficiency and safety, and its target is host Hsc70 instead of viral polymerase. Thus, natural products from traditional Chinese herbs will provide prolific candidates for searching new anti-HBV drugs with diverse mechanisms.

Hepatitis B as a type of chronic hepatitis is accompanied with high levels of alanine transaminase (ALT) and aspartate transaminase (AST). In addition to anti-HBV efficiency, TCMs can reduce the ALT and AST levels by improving the hepatic function of patients, and relieve HBV-associated clinical symptoms, e.g. anorexia, hypodynamia, nausea and vomiting, to improve the life quality of patients. Thus, TCMs maybe have advantages in treating HBV infection with multi-component and multi-target properties. Although, TCMs are fascinating sources for anti-HBV candidates, the complicated constituent and time-consuming isolation restrict the following drug development. Chemical modification on active natural products is an effective method in searching for anti-HBV agents, which can rapidly yield plenty of candidates from the starting substrates, and rationally increase the bioactivity, decrease the toxicity, or improve their physicochemical properties under the guidance of SARs.

## References

[1] The World Health Organization (WHO) (2015). Guidelines for the Prevention Care and Treatment of Persons with Chronic Hepatitis B Infection.

[2] Lavanchy D (2004). J. Viral Hepat..

[3] Wang GF, Shi LP, Zuo JP (2008). Virol. Sin..

[4] Kang L, Pan J, Wu J, Hu J, Sun Q, Tang J (2015). Viruses.

[5] Pu XF, Peng F (2012). Med. Recapitul..

[6] Geng CA, Wang LJ, Guo RH, Chen JJ (2013). Mini-Rev. Med. Chem..

[7] Wang LJ, Geng CA, Guo RH, Zhang Q, Chen JJ (2011). Asian Chem. Lett..

[8] Zhao Y, Geng CA, Sun CL, Ma YB, Huang XY, Cao TW, He K, Wang H, Zhang XM, Chen JJ (2014). Fitoterapia.

[9] Zhao Y, Geng CA, Ma YB, Huang XY, Chen H, Cao TW, He K, Wang H, Zhang XM, Chen JJ (2014). J. Ethnopharmacol..

[10] Zhao Y, Geng CA, Chen H, Ma YB, Huang XY, Cao TW, He K, Wang H, Zhang XM, Chen JJ (2015). Bioorg. Med. Chem. Lett..

[11] Geng CA, Yang TH, Huang XY, Yang J, Ma YB, Li TZ, Zhang XM, Chen JJ (2018). J. Ethnopharmacol..

[12] Geng CA, Chen XL, Huang XY, Ma YB, He K, Zhou NJ, Cao TW, Zhang XM, Chen JJ (2015). J. Ethnopharmacol..

[13] Romero MR, Efferth T, Serrano MA, Castano B, Macias RIR, Briz O, Marin JJG (2005). Antiviral Res..

[14] Zhao ZH, Wu F, Zheng LY, Wang LY, Hou YY, Geng N, Wang GF (2016). Chin. J. Exp. Tradit. Med. Form..

[15] Geng CA, Jiang ZY, Ma YB, Luo J, Zhang XM, Wang HL, Shen Y, Zuo AX, Zhou J, Chen JJ (2009). Org. Lett..

[16] Geng CA, Zhang XM, Shen Y, Zuo AX, Liu JF, Ma YB, Luo J, Zhou J, Jiang ZY, Chen JJ (2009). Org. Lett..

[17] Geng CA, Zhang XM, Ma YB, Jiang ZY, Luo J, Wang HL, Zhou J, Chen JJ (2010). Tetrahedron Lett..

[18] Geng CA, Wang LJ, Zhang XM, Ma YB, Huang XY, Luo J, Guo RH, Zhou J, Shen Y, Zuo AX, Jiang ZY, Chen JJ (2011). Chem-Eur. J..

[19] Geng CA, Zhang XM, Ma YB, Luo J, Chen JJ (2011). J. Nat. Prod..

[20] Geng CA, Zhang XM, Ma YB, Jiang ZY, Liu JF, Zhou J, Chen JJ (2010). J. Asian Nat. Prod. Res..

[21] Geng CA, Zhang XM, Ma YB, Huang XY, Chen JJ (2013). Nat. Prod. Bioprospect..

[22] Geng CA, Chen XL, Zhou NJ, Chen H, Ma YB, Huang XY, Zhang XM, Chen JJ (2014). Org. Lett..

[23] Wang HL, Cao TW, Jiang FQ, Geng CA, Zhang XM, Huang XY, Wang LJ, He K, Chen H, Liang WJ, Rong GQ, Chen JJ (2013). Tetrahedron Lett..

[24] Wang HL, Geng CA, Zhang XM, Ma YB, Jiang ZY, Chen JJ (2010). China J. Chin. Mater. Med..

[25] Wang HL, He K, Geng CA, Zhang XM, Ma YB, Luo J, Chen JJ (2012). Planta. Med..

[26] Wang HL, Geng CA, Ma YB, Zhang XM, Chen JJ (2013). Fitoterapia.

[27] He K, Ma YB, Geng CA, Zhang XM, Cao TW, Jiang FQ, Chen JJ (2011). Nat. Prod. Bioprospect..

[28] He K, Cao TW, Wang HL, Geng CA, Zhang XM, Chen JJ (2015). China J. Chin. Mater. Med..

[29] Geng JL, Geng CA, Chen JJ (2012). Nat. Prod. Res. Dev..

[30] Jie XX, Geng CA, Huang XY, Ma YB, Zhang XM, Zhang RP, Chen JJ (2015). Fitoterapia.

[31] He K, Ma YB, Cao TW, Wang HL, Jiang FQ, Geng CA, Zhang XM, Chen JJ (2012). Planta. Med..

[32] He K, Cao TW, Wang HL, Geng CA, Zhang XM, Chen JJ (2015). China J. Chin. Mater. Med..

[33] Cao TW, Geng CA, Jiang FQ, Ma YB, He K, Zhou NJ, Zhang XM, Zhou J, Chen JJ (2013). Fitoterapia.

[34] Geng CA, Chen XL, Huang XY, Ma YB, He K, Zhou NJ, Cao TW, Zhang XM, Chen JJ (2015). Tetrahedron Lett..

[35] Cao TW, Geng CA, Ma YB, He K, Wang HL, Zhou NJ, Zhang XM, Tao YD, Chen JJ (2013). Planta. Med..

[36] Cao TW, Geng CA, Ma YB, Zhang XM, Zhou J, Tao YD, Chen JJ (2015). Fitoterapia.

[37] Cao TW, He K, Zhang XM, Zhou J, Chen JJ (2016). Chin. J. Org. Chem..

[38] He K, Cao TW, Wang HL, Geng CA, Zhang XM, Chen JJ (2015). China J. Chin. Mater. Med..

[39] He K, Geng CA, Cao TW, Wang HL, Ma YB, Zhang XM, Chen JJ (2016). J. Asian Nat. Prod. Res..

[40] Zhou NJ, Geng CA, Huang XY, Ma YB, Zhang XM, Wang JL, Chen JJ (2015). Fitoterapia.

[41] Jiang FQ, Zhang XM, Ma YB, Geng CA, Jiang ZY, Chen JJ (2011). China J. Chin. Mater. Med..

[42] Cao TW, Geng CA, Ma YB, He K, Zhou NJ, Zhou J, Zhang XM, Chen JJ (2015). China J. Chin. Mater. Med..

[43] Wang HL, Chen H, Geng CA, Zhang XM, Chen JJ (2011). J. Yunnan Minzu Univ..

[44] Wang HL, Chen H, Geng CA, Zhang XM, Ma YB, Jiang ZY, Chen JJ (2011). China J. Chin. Mater. Med..

[45] Jiang ZY, Zhang XM, Zhang FX, Liu N, Zhao F, Zhou J, Chen JJ (2006). Planta Med..

[46] Jiang ZY, Zhang XM, Zhou J, Zhang FX, Chen JJ, Lu Y, Wu L, Zheng QT (2007). Chem. Pharm. Bull..

[47] Chiang LC, Ng LT, Liu LT, Shieh DE, Lin CC (2003). Planta Med..

[48] Xu HB, Ma YB, Huang XY, Geng CA, Wang H, Zhao Y, Yang TH, Chen XL, Yang CY, Zhang XM, Chen JJ (2015). J. Ethnopharmacol..

[49] Cheng P, Ma YB, Yao SY, Zhang Q, Wang EJ, Yan MH, Zhang XM, Zhang FX, Chen JJ (2007). Bioorg. Med. Chem. Lett..

[50] Liu JF, Jiang ZY, Zhang Q, Shi Y, Ma YB, Xie MJ, Zhang XM, Chen JJ (2010). Planta Med..

[51] Liu JF, Zhang XM, Shi Y, Jiang ZY, Ma YB, Chen JJ (2010). China J. Chin. Mater. Med..

[52] Liu JF, Jiang ZY, Geng CA, Zhang Q, Shi Y, Ma YB, Zhang XM, Chen JJ (2011). Chem. Biodivers..

[53] Geng CA, Yao SY, Xue DQ, Zhang XM, Jiang ZY, Ma YB, Chen JJ (2010). China J. Chin. Mater. Med..

[54] Geng CA, Ma YB, Zhang XM, Yao SY, Xue DQ, Zhang RP, Chen JJ (2012). J. Agric. Food Chem..

[55] Yan MH, Cheng P, Jiang ZY, Ma YB, Zhang XM, Zhang FX, Liu MY, Zheng YT, Chen JJ (2008). J. Nat. Prod..

[56] Liu WF, Jiang ZY, Zhang XM, Ma YB, Chen JJ (2009). China J. Chin. Mater. Med..

[57] Liu WF, Jiang ZY, Zhang XM, Ma YB, Chen JJ (2009). China J. Chin. Mater. Med..

[58] Jiang ZY, Liu WF, Zhang XM, Luo J, Ma YB, Chen JJ (2013). Bioorg. Med. Chem. Lett..

[59] Shin MS, Kang EH, Lee YI (2005). Antiviral Res..

[60] Lv JJ, Wang YF, Zhang JM, Yu S, Wang D, Zhu HT, Cheng RR, Yang CR, Xu M, Zhang YJ (2014). Org. Biomol. Chem..

[61] Wang SG, Li JY, Huang H, Gao W, Zhuang CL, Li B, Zhou P, Kong DY (2009). Biol. Pharm. Bull..

[62] Sun Y, Li LJ, Li J, Li Z (2007). Virol Sin..

[63] Geng CA, Huang XY, Lei LG, Zhang XM, Chen JJ (2012). Chem. Biodivers..

[64] Geng CA, Chen H, Chen XL, Huang XY, Lei LG, Chen JJ (2014). Int. J. Mass Spectrom..

[65] Guo QL, Zhao L, You QD, Yang Y, Gu HY, Song GL, Lu N, Xin J (2007). Antiviral Res..

[66] Ding PL, Liao ZX, Huang H, Zhou P, Chen DF (2006). Bioorg. Med. Chem. Lett..

[67] Ding PL, Huang H, Zhou P, Chen DF (2006). Planta Med..

